# Crosstalk between acetylation and the tyrosination/detyrosination cycle of α-tubulin in Alzheimer’s disease

**DOI:** 10.3389/fcell.2022.926914

**Published:** 2022-08-26

**Authors:** José Martínez-Hernández, Julie Parato, Aditi Sharma, Jean-Marc Soleilhac, Xiaoyi Qu, Ellen Tein, Andrew Sproul, Annie Andrieux, Yves Goldberg, Marie-Jo Moutin, Francesca Bartolini, Leticia Peris

**Affiliations:** ^1^ Inserm, U1216, CEA, CNRS, Grenoble Institut Neurosciences, Université Grenoble Alpes, Grenoble, France; ^2^ Department of Pathology and Cell Biology, Columbia University Irving Medical Center, New York, NY, United States; ^3^ Department of Natural Sciences, SUNY Empire State College, Brooklyn, NY, United States; ^4^ Taub Institute for Research Alzheimer’s Disease and the Aging Brain, Columbia University Irving Medical Center, New York, NY, United States

**Keywords:** tubulin, microtubule, acetylation, tyrosination, neuron, Alzheimer’s disease, tubulin tyrosine ligase

## Abstract

Microtubules (MTs) support a variety of neuronal functions, such as maintenance of cell structure, transport, and synaptic plasticity. Neuronal MTs are highly heterogeneous due to several tubulin isotypes and the presence of multiple post-translational modifications, such as detyrosination and acetylation. The tubulin tyrosination/detyrosination cycle is a key player in the maintenance of MT dynamics, as tyrosinated tubulin is associated with more dynamic MTs, while detyrosinated tubulin is linked to longer lived, more stable MTs. Dysfunction of tubulin re-tyrosination was recently correlated to Alzheimer’s disease progression. The implication of tubulin acetylation in Alzheimer’s disease has, however, remained controversial. Here, we demonstrate that tubulin acetylation accumulates in post-mortem brain tissues from Alzheimer’s disease patients and human neurons harboring the Alzheimer’s familial APP-V717I mutation. We further show that tubulin re-tyrosination, which is defective in Alzheimer’s disease, can control acetylated tubulin in primary neurons irrespective of the levels of the enzymes regulating tubulin acetylation, suggesting that reduced MT dynamics associated with impaired tubulin re-tyrosination might contribute to the accumulation of tubulin acetylation that we detected in Alzheimer’s disease.

## Introduction

The MT cytoskeleton in neurons supports overall neuronal function, including learning and memory processes (Uchida et al., 2014). Dimers of α- and β-tubulin polymerize to form MTs, which are comprised of 13 protofilaments organized around a hollow core. MTs can be stable or dynamic, with dynamic MTs going through periods of growth and disassembly.

Tubulin can acquire a large variety of rapid and reversible post-translational modifications that label distinct MT subpopulations in neurons and are expected to program them for specific functions (Moutin et al., 2021; Parato and Bartolini, 2021; Waites et al., 2021). These post translational modifications act as a code, affecting binding of MT associated proteins and motor proteins as well as MT stability (Janke and Magiera, 2020). For example, tyrosinated tubulin modulates binding of MT plus end tracking proteins, kinesins, and cytosolic dynein (Peris et al., 2009; Janke and Magiera, 2020). Furthermore, while tyrosinated tubulin is associated with dynamic MTs, detyrosinated and Δ2 tubulins are associated with more stable, longer-lived MTs (Kreis, 1987; Paturle-Lafanechere et al., 1994). The tyrosination/detyrosination (Tyr/deTyr) cycle is regulated by the vasohibin-small vasohibin binding protein complexes (VASH1/2-SVBP) and MATCAP, which remove the terminal tyrosine from the α-tubulin C-terminal tail, while tubulin tyrosine ligase (TTL) re-adds it. VASH-SVBP and MATCAP act on MTs, while TTL acts on soluble tubulin dimers (Kreis, 1987; Webster et al., 1987; Prota et al., 2013; Aillaud et al., 2017; Nieuwenhuis et al., 2017; Landskron et al., 2022). The pool of detyrosinated tubulin can be further processed by cytosolic carboxy peptidases (CCPs) from the deglutamylase family, that remove the terminal glutamate, turning detyrosinated into Δ2 tubulin (Rogowski et al., 2010). As TTL cannot tyrosinate Δ2 tubulin, this modification is considered as irreversible, withdrawing Δ2 tubulin from the Tyr/deTyr cycle (Prota et al., 2013). Interestingly, we recently observed that in Alzheimer’s disease (AD), TTL levels drop, driving MTs towards accumulating detyrosinated and Δ2 tubulin, markers of MT longevity and inhibitors of synaptic plasticity by reducing the frequency of tyrosinated MT invasion into dendritic spines (Peris et al., 2022).

Tubulin is acetylated on several residues but acetylation of lysine 40 (K40) on α-tubulin is a unique post translational modification, as it is located in the inner lumen of the MT (L'Hernault and Rosenbaum, 1985). In cells, α-tubulin acetylation accumulates on long lived MTs and on MTs stabilized by taxanes (Piperno et al., 1987). αK40 acetylation is positively regulated by α-TAT1 (acetyl transferase α-Tat1) and reversed by the enzymes histone deacetylase family member 6 (HDAC6) and sirtuin 2 (SIRT2) (Hubbert et al., 2002; North et al., 2003). α-TAT1 is specific for tubulin while the deacetylases act on diverse substrates. α-TAT1 works on MTs over free tubulin and has a moderate catalytic rate, allowing it to act as a slow marker of MT aging (Szyk et al., 2014). HDAC6 can deacetylate a diverse type of substrates to carry out essential functions in a large number of cell signaling pathways. As a further confounder, Tau can act as an inhibitor of HDAC6 activity, thereby limiting its wide assortment of functions (Perez et al., 2009). In addition, HDAC6 can indirectly regulate MT dynamics by the interaction with the plus end-tracking protein EB1 and a dynactin core component Arp1, with cytoplasmic dynein, and with formin homology proteins mDia2 and mDia1 (Kawaguchi et al., 2003; Destaing et al., 2005; Bershadsky et al., 2006; Zilberman et al., 2009).

Acetylated α-tubulin is involved both in dendrite and axon morphogenesis. Loss of α-tubulin acetylation predisposes neurons to axon over-branching and excessive growth (Jenkins et al., 2017; Dan et al., 2018). The contribution of acetylated tubulin to neuronal morphogenesis was related to molecular effectors such as CAMSAP3 (calmodulin-regulated spectrin-associated protein 3) which preferentially associates with non-acetylated MTs to regulate MT minus-end dynamics (Pongrakhananon et al., 2018) or to MT severing by katanin that preferentially occurs at acetylated MT sites in dendrites (Sudo and Baas, 2010).

While tubulin acetylation and detyrosination occur at different locations of the MT wall and are driven by different regulatory enzymes, both post-translational modifications are frequently observed in the same sub-population of stable MTs. Indeed, combining super-resolution techniques and expansion microscopy, two distinct pools of MTs were recently shown to form non-overlapping bundles of opposite orientation in dendrites: a stable core enriched in acetylated and Δ2 tubulins, and an outer shell composed of dynamic tyrosinated MTs (Katrukha et al., 2021). This organization is believed to enhance the specific recruitment of plus-end directed kinesin-1 motors to stable MT bundles (Balabanian et al., 2017), whereas outer dynamic tyrosinated MTs might favor the loading of minus-end oriented dynein (McKenney et al., 2016; Nirschl et al., 2016).

A correlation between tubulin acetylation and detyrosination was also recently observed in α-TAT1 acutely depleted and knock-out (KO) murine embryonic fibroblasts, in which acetylated and detyrosinated MTs appeared to both be reduced (Xu et al., 2017). As an enzymatic overlap between these tubulin modifications is unlikely, their co-existence on the same MT could be a consequence of an increase in MT stability. In the case of acetylation, the addition of the acetyl group limits the range of motion of the αK40 loop, weakens lateral contacts, and allows for increased MT flexibility, resulting in higher resistance of MTs to mechanical stress. Consequently, acetylation prevents MT breakage, thus further prolonging MT longevity on a previously stabilized MT (Portran et al., 2017; Xu et al., 2017; Eshun-Wilson et al., 2019). Unlike acetylation that directly promotes MT longevity, detyrosination and Δ2 do not directly modify MT dynamics (Lafanechere and Job, 2000), but either modification can increase MT stability by reducing motor-driven MT depolymerization (Peris et al., 2009).

MT acetylation has long received attention in the context of AD, a condition in which a subset of neurons residing in vulnerable regions of the brain progressively degenerate. However, studies addressing the level of tubulin acetylation in post mortem human brain samples have yielded divergent results. It has been reported that acetylated tubulin decreases in AD (Hempen and Brion, 1996; Silva et al., 2017), correlating with a higher abundance of HDAC6 (Ding et al., 2008). In contrast, other studies suggest that even though the total MT mass diminishes in AD, the proportion of tubulin that is acetylated is actually increased (Perez et al., 2009; Zhang et al., 2015). Using cellular and transgenic models of AD, HDAC6 was found to promote aspects of the AD phenotype, and this was proposed to occur *via* HDAC6 interaction with Tau (Govindarajan et al., 2013; Zhang L. et al., 2014; Tseng et al., 2017). On the other hand, HDAC6 appears to be inhibited by oligomeric Aβ (oAβ) peptide (Tsushima et al., 2015) and cultured neurons treated with oAβ accumulate acetylated tubulin (Qu et al., 2017).

Herein, we re-visit the issue of tubulin acetylation in AD by analyzing a series of well-staged brain samples obtained after a consistently short post-mortem interval, and we examine the functional crosstalk between acetylation and the tyrosination/detyrosination cycle of α-tubulin in AD. We chose to examine the tyrosination/detyrosination cycle as a potential driver of α-tubulin acetylation because we recently showed a decrease of TTL and an increase in detyrosinated and Δ2 tubulin levels in sporadic and familial forms of AD. Consistent with an increase in MT stability, MTs in hiPSC-derived human neurons bearing the AD-causing London (V717I) APP mutation also had longer growth rates and fewer catastrophes (Peris et al., 2022). We used the same series of brain samples in this work, that is larger than in previously published studies and includes four well-defined brain areas for each subject. We combined these data with semi-quantitative immunostaining of brain sections from a different set of control and diseased individuals and with analysis of hiPSC-derived human neurons bearing the London mutation. In all of these three settings where we previously showed an increase of non-tyrosinated tubulin, we found that AD also correlates with a rise in acetylated tubulin while we observed no correlation in the amounts of HDAC6 or α-TAT1. To understand the origin of the excess of tubulin acetylation, we examined the functional cross-talk between acetylation and tyrosination/detyrosination cycle of α-tubulin in neurons. By depleting TTL or SVBP (or acute VASH-SVBP inactivation) in cultured rodent neurons we could, respectively, enhance or reduce tubulin acetylation levels. We propose that the loss of tubulin re-tyrosination we recently showed in AD could provide an increase in MT stability that accounts for higher levels of tubulin acetylation, resulting in amplification of the pathological phenotype.

## Results

We recently reported that tubulin re-tyrosination was deficient in AD and correlated this phenotype to loss of MT dynamics in human neurons bearing a mutation underlying a familial form of AD (Peris et al., 2022). Herein, we sought to determine whether this MT phenotype further correlated with a change in the acetylation of lysine 40 on α-tubulin, a tubulin post-translational modification associated with an increase in MT stability, and explored whether we could affect MT acetylation by interfering with the tubulin tyrosination/detyrosination cycle.

### Enhanced acetylation of neuronal microtubules in Alzheimer’s disease

**FIGURE 1:**
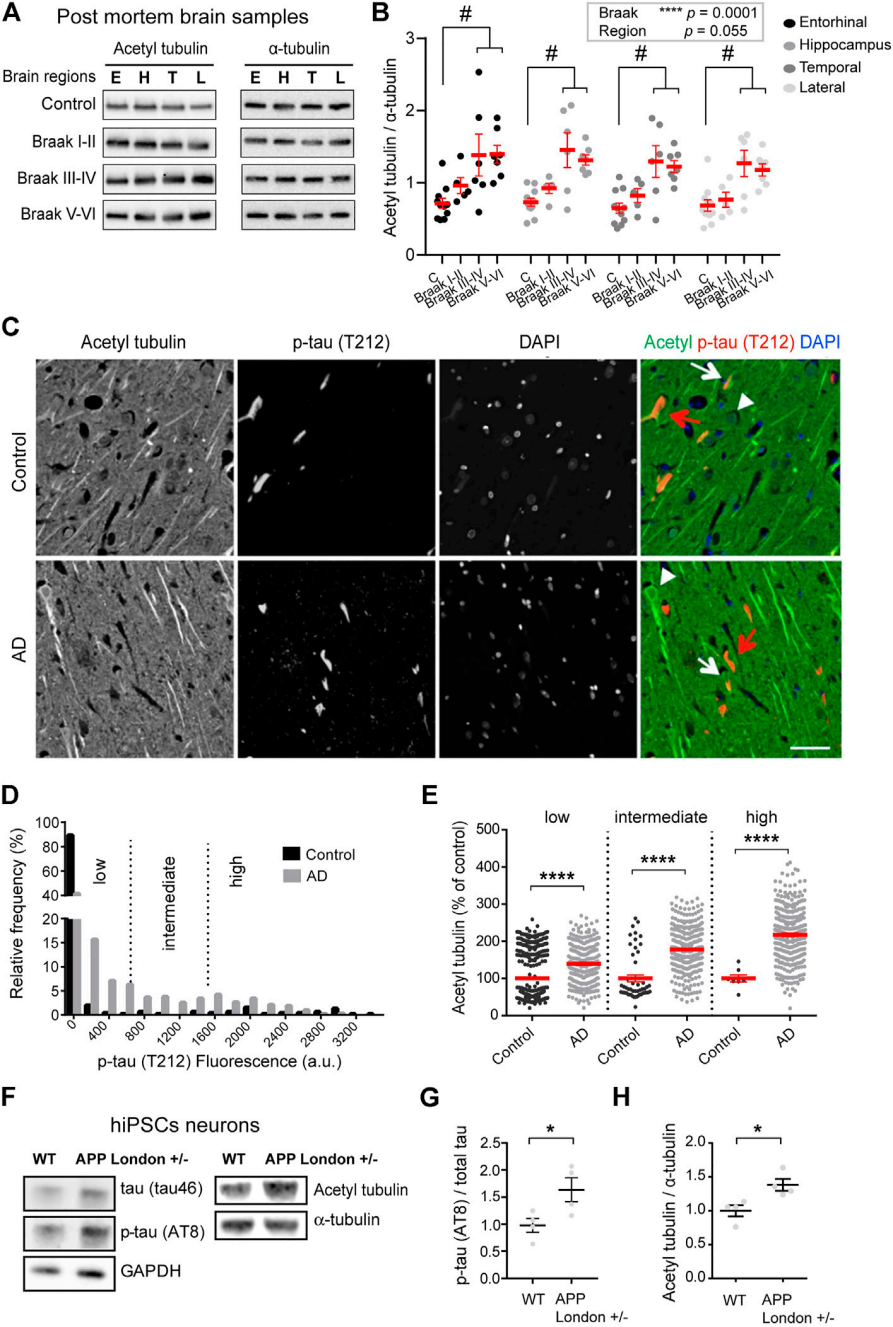
Increased acetylated α-tubulin correlates with phospho-tau accumulation in AD. **(A)** Representative immunoblots of acetylated (acetyl) tubulin and total α-tubulin levels in brain homogenates from entorhinal cortex (E), hippocampus (H), temporal (T), and lateral prefrontal cortex (L) from control, early AD (Braak I-II), middle AD (Braak III-IV), and late AD (Braak V-VI) patients. **(B)** Quantification of the ratio of acetylated tubulin and total α-tubulin levels in each brain region from control and AD patients. An internal standard corresponding to a WT sample was used for normalization and considered as 100% and the values for each unknown sample was calculated as a % of this standard (see *Material and methods*). Each dot in the graph represents the mean of a triplicate analysis of one sample. The mean ± SEM of these dot values is shown for each factor combination. The dependence of protein levels on, respectively, clinical stage and brain area was quantitated in each case using a linear mixed model, with Braak stage and brain region as fixed effect factors. Boxed *p* values measure the overall significance of these factors (type II Wald *F* test of model coefficients). In each brain area, post-hoc testing of variations due to individual Braak stages was performed by Dunnett’s test of differences with control. Significance levels are indicated as #*p* < 0.05 for control and middle and late Braak stages from AD patient brains. *n* = 11, 5, 6, and 7 brain samples for control, Braak I-II, Braak III-IV, and Braak V-VI, respectively. **(C)** Dual immunostaining of acetylated α-tubulin and T212-reactive phospho-tau, combined with nuclear staining with DAPI, was performed on sections of control (upper panel) and AD patient hippocampi (lower panel). Neurons with low (white arrowheads), intermediate (white arrows) or high (red arrows) levels of T212 immunofluorescence are shown. Scale bar: 50 µm. **(D)** Relative frequency distribution of phospho-tau (T212) immunofluorescence levels (arbitrary units) in pyramidal neurons of control and AD brains. Low, intermediate, and high phospho-tau groups were defined based on fluorescence intensity. Two-sample Kolmogorov-Smirnov test, ****p < 0.0001. **(E)** Intensity of acetylated α-tubulin immunofluorescence in pyramidal cell bodies of AD hippocampal neurons relative to mean of values of control, shown as a function of T212 labelling level. Mean ± SEM. *n* = 397, 53, and 8 neurons for controls and *n* = 290, 325, and 330 for AD neurons in low, intermediate and high phospho-tau groups, respectively. Among AD neurons, both the variation due to the change in T212 level (Kruskal-Wallis test, *p* < 0.0001) and the effect of AD status within each T212 level (Kruskal-Wallis test, ****p < 0.0001) are significant. **(F)** Immunoblots of phospho-specific tau (AT8), total tau (tau46), acetylated α-tubulin, and total α-tubulin in neurons bearing the APP-London mutation or isogenic controls. **(G,H)** Immunoblot quantifications of phospho-tau normalized to total tau and acetylated tubulin to total α-tubulin. Graphs represent mean ± SEM. *n* = 4 independent neuronal differentiation experiments. Unpaired *t*-test, *p < 0.05.

A series of post mortem brain samples from patients at different stages of AD (as assessed by histo-pathological scoring according to Braak) or age-matched, non-demented controls was collected as previously described (Peris et al., 2022) (Supplementary Table S1). For each individual, we analyzed samples from four brain areas known to be successively affected by AD: entorhinal cortex, hippocampus, lateral frontal cortex, and temporal cortex as used in Peris et al. (2022). Equal amounts of total brain protein from all samples were analyzed by immunoblotting, using antibodies raised against acetylated or total tubulin. Blots were standardized by normalizing band intensities with respect first to total protein in the corresponding lanes, and then to a control sample loaded on the same gel (Figure 1A).

Statistical modeling of the data indicated that the acetylated vs. total tubulin ratio significantly varied as a function of the disease state [F(3,25) = 10.63, *p* = 0.0001, Wald *F* test of linear mixed model]. For all four brain areas, the mean ratio of acetylated vs. total tubulin in late AD brain (Braak V-VI) was about double that in non-demented control (Figure 1B). Post hoc tests in individual areas showed the increase to be statistically significant in all four regions (Supplementary Table S2).

Interestingly, in all four regions, up-regulation of tubulin acetylation was detectable already at early AD stages (134.5%, 126.5%, 126.8%, and 111.6% of acetylated tubulin in entorhinal, hippocampus, lateral prefrontal, and temporal cortex, respectively, for Braak I-II patients compared to non-demented individuals) and became statistically significant at middle stages (193.05%, 199.0%, 199.5%, and 185.0% of acetylated tubulin in entorhinal, hippocampus, lateral prefrontal, and temporal cortex, respectively, of Braak III-IV patients compared to non-demented individuals) These biochemical analyses provide evidence that acetylated α-tubulin accumulation correlates to AD progression.

To confirm these results at the single neuron level, we performed semi-quantitative, dual-channel immunofluorescence microscopy of acetylated tubulin and phosphorylated Tau in hippocampal sections from AD and control (non-demented) individuals (Supplementary Table S3). The phosphorylated form of Tau, labeled with antibody T212, was used as a marker of pathological Tau aggregates. As expected, the frequency of neurons with elevated phospho-Tau fluorescence was much higher in AD than in control hippocampus, as shown by a strong rightward shift in the fluorescence intensity distribution (Figures 1C,D). However, a large proportion of AD neurons still had phospho-Tau levels at the lower end of the range, presumably being at an early stage of degeneration. To distinguish between neuronal subsets at different stages of degeneration, we divided the phospho-Tau fluorescence scale into three ranges (low, medium and high) of labeling intensity. We then calculated for each neuron the ratio of its acetylated tubulin fluorescence value to the mean acetylated tubulin value of control neurons that were within the same phospho-Tau range. The results show that for all three classes of phospho-Tau abundance, the mean level of acetylated tubulin is significantly higher in AD neurons than in their non-AD counterparts (Figure 1E, 139.70% + 4.35%, 177.60% + 3.24%, and 217.50% + 4.99% of acetylated tubulin in low, medium and high phospho-tau neurons from AD hippocampus, respectively). Remarkably, acetylated tubulin accumulated progressively as a function of phospho-Tau abundance in AD neurons (Kruskal-Wallis test, *p* < 0.0001), suggesting abnormal MT regulation in pathological conditions. Overall, the immunostaining data agree with the biochemical analysis above and suggest that excess tubulin acetylation occurs at an early step of AD pathogenesis.

To determine whether the increase of tubulin acetylation was a hallmark of familial AD, we first turned to hiPSC-derived human neurons harboring the AD-linked London mutation (V717I) into one allele of the APP gene (APP^London^), a well-characterized genetic driver of familial AD (Sun et al., 2019; Peris et al., 2022). We differentiated APP^London^ and APP^WT^ isogenic controls into human cortical neurons for 30–40 days *in vitro*, neurons were lysed and the relative levels of acetylated and total α-tubulin levels were analyzed by immunoblot. At this stage of differentiation, the mutant neurons accumulated tau protein, which was hyperphosphorylated (AT8, Figures 1F,G), confirming the occurrence of a previously described pathological feature associated with this APP mutation (Muratore et al., 2014). In agreement with the patient data, the ratio of acetylated to total α-tubulin was significantly higher in APP^London^-carrying neurons than in APP^WT^ isogenic controls (Figures 1F,H, 100.00% + 8.21% and 138.00% + 8.87% for APP^WT^ and APP^London^, respectively).

To evaluate any change in the amount of the enzymes responsible for tubulin acetylation, we probed the same samples with antibodies raised against HDAC6 and α-TAT1. In the *post mortem* brain samples from patients at different stages of AD, we found no significant relationship between disease stages and HDAC6 amounts [F(3,25) = 0.76, *p* = 0.53, Wald *F* test] (Supplementary Figures S1A,B). In the hiPSC neuronal lysates, however, we found that the APP mutation induced a large increase in HDAC6 levels (Supplementary Figure S1D, 100.00% + 22.19% and 388.10% + 47.50% for APP^WT^ and APP^London^, respectively), while the level of α-TAT1 remained unchanged (Supplementary Figure S1E, 100.00% + 17.24% and 108.00% + 11.29% for APP^WT^ and APP^London^, respectively). Therefore, the enhanced acetylation of α-tubulin occurring in AD cannot be attributed to a loss of the deacetylase HDAC6, or to a gain of the acetylase α-TAT1.

### Tubulin tyrosine ligase depletion is sufficient to increase α-tubulin acetylation

**FIGURE 2:**
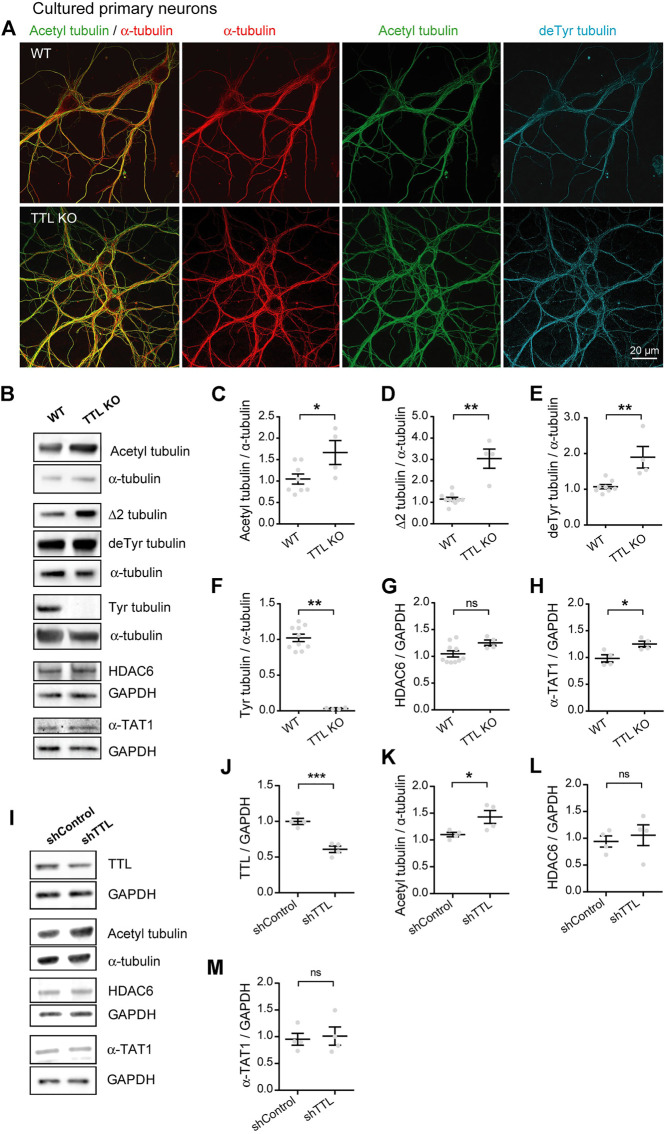
Increased acetylated tubulin levels in cultured primary neurons with TTL null or reduced TTL levels. **(A)** Confocal images showing representative examples of mouse cultured hippocampal neurons at DIV17 from WT and TTL KO embryos, stained for total α-tubulin (red), acetylated tubulin (acetyl, green), and detyrosinated tubulin (deTyr, blue). Scale bar: 20 µm. **(B)** Immunoblot analysis of acetylated tubulin, Δ2 tubulin, detyrosinated tubulin, tyrosinated tubulin, total α-tubulin, HDAC6, α-TAT1, and GAPDH from lysates of mouse cultured cortical neurons at DIV15 from WT and TTL KO embryos. Immunoblot quantifications of the ratio of acetylated tubulin **(C)**, Δ2 tubulin **(D)**, detyrosinated tubulin **(E)** and tyrosinated tubulin **(F)** to total α-tubulin, and the ratio of HDAC6 **(G)** and α-TAT1 **(H)** to GAPDH. Data represent mean ± SEM. *n* = 10 and 4 independent neuronal differentiation experiments for WT and TTL KO, respectively. Unpaired *t* test for **(C,E,G)**, Mann Whitney test for the others, ns, not significant, *p < 0.05 and **p < 0.01. **(I)** Immunoblot analysis of TTL, acetylated tubulin, α-tubulin, HDAC6, α-TAT1 and GAPDH from lysates of rat cultured primary hippocampal neurons at DIV21 infected with shTTL and their control at DIV17. GAPDH was used for normalization of TTL, HDAC6 and α-TAT1; and total α-tubulin for acetylated tubulin. Immunoblot quantifications of the ratio of TTL/GAPDH **(J)**, acetylated/total α-tubulin **(K)**, HDAC6/GAPDH **(L)** and α-TAT1/GAPDH **(M)**. Data represent mean ± SEM. *n* = 4 independent neuronal differentiation experiments. Unpaired *t*-test, ns, not significant, *p < 0.05, ***p < 0.001.

The data above fail to support the possibility that the increase in acetylated tubulin in AD could result from changes in the levels of the enzymes regulating acetylation. Tubulin acetylation is a marker of longer lived MTs and often correlates with tubulin detyrosination, which we recently showed to be increased in AD alongside a deficit in TTL levels (Peris et al., 2022). Furthermore, while tubulin detyrosination typically labels stabilized and poorly dynamic MTs, it can also confer MT resistance by inhibiting binding of depolymerizing kinesins (Kreis, 1987; Khawaja et al., 1988; Infante et al., 2000; Peris et al., 2009). Here, we investigated whether loss of TTL alone and deriving accumulation of detyrosinated tubulin could also increase tubulin acetylation in primary neurons. For this, primary cultures of hippocampal neurons derived from TTL KO mouse embryos or wild-type controls were used to perform triple immunofluorescent staining with antibodies raised against total (red), acetylated (green), and detyrosinated (blue) α-tubulin (Figure 2A). We observed an enhanced proportion of acetylated and detyrosinated MTs in TTL KO neurons compared to wild-type cells. Immunoblot analysis of cortical neurons from the same embryos showed that TTL KO neurons were nearly devoid of tyrosinated tubulin (Figures 2B,F, 102.20% + 5.18% and 3.33% + 0.56% for WT and TTL KO, respectively), indicating a dramatic drop in dynamic MTs. Conversely, the amounts of detyrosinated and Δ2 tubulin were strongly elevated, in agreement with immunofluorescence data (Figures 2B,D,E, 107.20% + 5.94% and 189.80% + 30.18% of detyrosinated tubulin levels for WT and TTL KO, respectively; and 115.30% + 8.29% and 304.00% + 44.89% of Δ2 tubulin levels for WT and TTL KO, respectively). Quantification of the blots indicated that the mean proportion of acetylated MTs was increased by more than 50% in the TTL KO neurons compared to wild-type controls (Figures 2B,C, 104.80 + 11.80 and 166.60 + 27.90 for WT and TTL KO, respectively). Enhanced MT acetylation was associated with no significant change in HDAC6 level and a slight increase in the level of α-TAT1 (Figures 2B,G,H, 104.60% + 5.72% and 125.20% + 5.35% of HDAC6 levels for WT and TTL KO, respectively; 98.38% + 6.89% and 125.20% + 5.35% of α-TAT1 levels for WT and TTL KO, respectively). These results indicate that TTL loss causes neurons to accumulate detyrosinated, stable MTs that become heavily acetylated.

Since complete TTL loss is unlikely to occur in AD and TTL depletion in a mouse model could result in compensation and/or confounding effects on neuronal development, we also studied neurons which were only partially depleted in TTL. To do this, we infected cultured wild-type neurons with a TTL-targeting or control shRNA (sh) vector and analyzed the amounts of acetylated tubulin and its regulatory enzymes by immunoblotting. TTL depletion, which resulted in a 40% drop in TTL levels (Figures 2I,J, 100.00% + 4.25% and 60.92% + 4.56% for shControl shRNA and shTTL shRNA, respectively), induced a significant increase in the acetylated vs. total tubulin ratio (Figures 2I,K, 110.10% + 4.15% and 142.90% + 12.00% for shControl and shTTL, respectively). By contrast, the levels of both HDAC6 and α-TAT1 remained unaffected (Figures 2I,L,M, 94.06% + 10.42% and 105.80% + 19.29% of HDAC6 levels for shControl and shTTL, respectively; 95.36% + 11.13% and 101.30% + 17.04% of α-TAT1 levels for shControl and shTTL, respectively). Thus, the rise in MT acetylation is observed following even a partial loss of TTL (as seen in AD) and independently of possible developmental compensatory processes.

### SVBP depletion is sufficient to reduce microtubule acetylation

If the heightened tubulin acetylation observed in TTL-lacking neurons directly results from the accumulation of stable detyrosinated MTs (Khawaja et al., 1988; Peris et al., 2009), then conversely blocking tubulin detyrosination would be expected to diminish acetylated tubulin levels.

**FIGURE 3:**
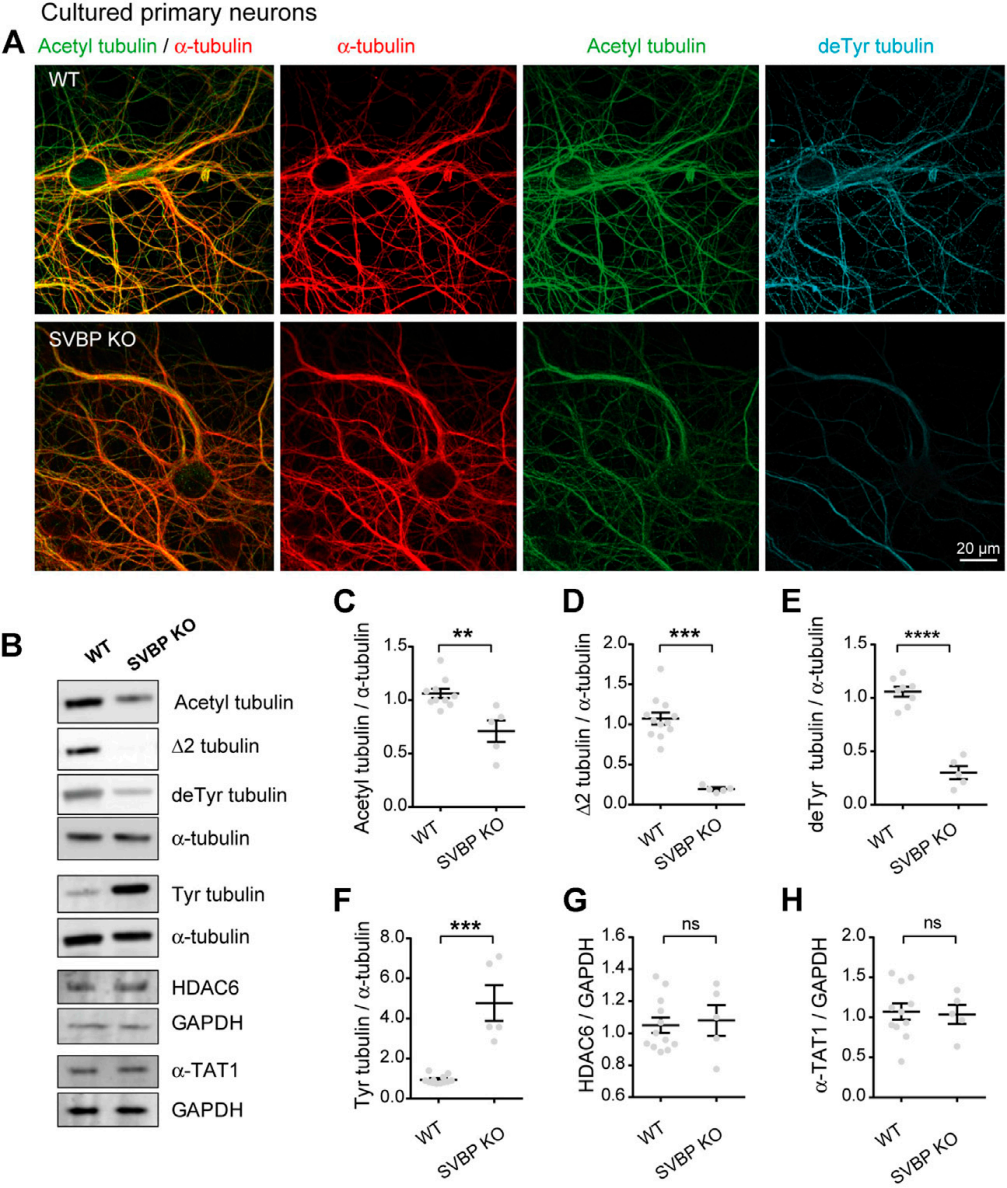
Decreased acetylated tubulin levels in cultured primary neurons with no detyrosination activity. **(A)** Confocal images showing representative examples of mouse cultured hippocampal neurons at DIV17 from WT and SVBP KO embryos, stained for total α-tubulin (red), acetylated tubulin (acetyl, green), and detyrosinated tubulin (deTyr, blue). Scale bar: 20 µm. **(B)** Immunoblot analysis of acetylated tubulin, Δ2 tubulin, detyrosinated tubulin, tyrosinated tubulin, total α-tubulin, HDAC6, α-TAT1, and GAPDH from lysates of mouse cultured cortical neurons at DIV15 from WT and SVBP KO embryos. Immunoblot quantifications of the ratio of acetylated tubulin **(C)**, Δ2 tubulin **(D)**, detyrosinated tubulin **(E)** and tyrosinated tubulin **(F)** to total α-tubulin, and the ratio of HDAC6 **(G)** and α-TAT1 **(H)** to GAPDH. Data represent mean ± SEM. *n* = 11 and 5 independent neuronal differentiation experiments for WT and SVBP KO, respectively. Mann Whitney test for **(D,F)**, Unpaired *t* test for the others, ns, not significant, **p < 0.01, ***p < 0.001 and ****p < 0.0001.

We tested this prediction in cultured neurons derived from SVBP KO mice, which we previously showed to be defective for tubulin detyrosination (Pagnamenta et al., 2019). Immunofluorescent staining and confocal microscopy revealed that in these neurons, the subset of acetylated MTs was strikingly reduced, along with that of detyrosinated MTs (Figure 3A). We confirmed this result by quantitating immunoblots prepared from parallel cultures of SVBP KO and wild-type cortical neurons. As expected, the changes in modified tubulins were inverse of those measured in TTL KO neurons, with a dramatic fall of detyrosinated and especially Δ2 tubulin (Figures 3B,D,E, 105.90% + 4.53% and 29.99% + 6.03% of detyrosinated tubulin for WT and SVBP KO, respectively 107.40% + 7.42% and 19.23 + 1.72% of Δ2 tubulin, for WT and SVBP KO, respectively), a 5-fold gain in tyrosinated tubulin (Figures 3B,F, 93.83 + 5.85 and 477.10 + 88.86 for WT and SVBP KO, respectively), and a significant drop in acetylated tubulin (Figures 3B,C, 106.20 + 4.31 and 71.00 + 10.12 for WT and SVBP KO, respectively). In contrast, HDAC6 and α-TAT1 were unchanged (Figures 3B,G,H, 104.90 + 4.76 and 108.00 + 9.63 for HDAC6; 107.30 + 10.18 and 103.80 + 11.94 for α-TAT1, for WT and SVBP KO, respectively).

**FIGURE 4:**
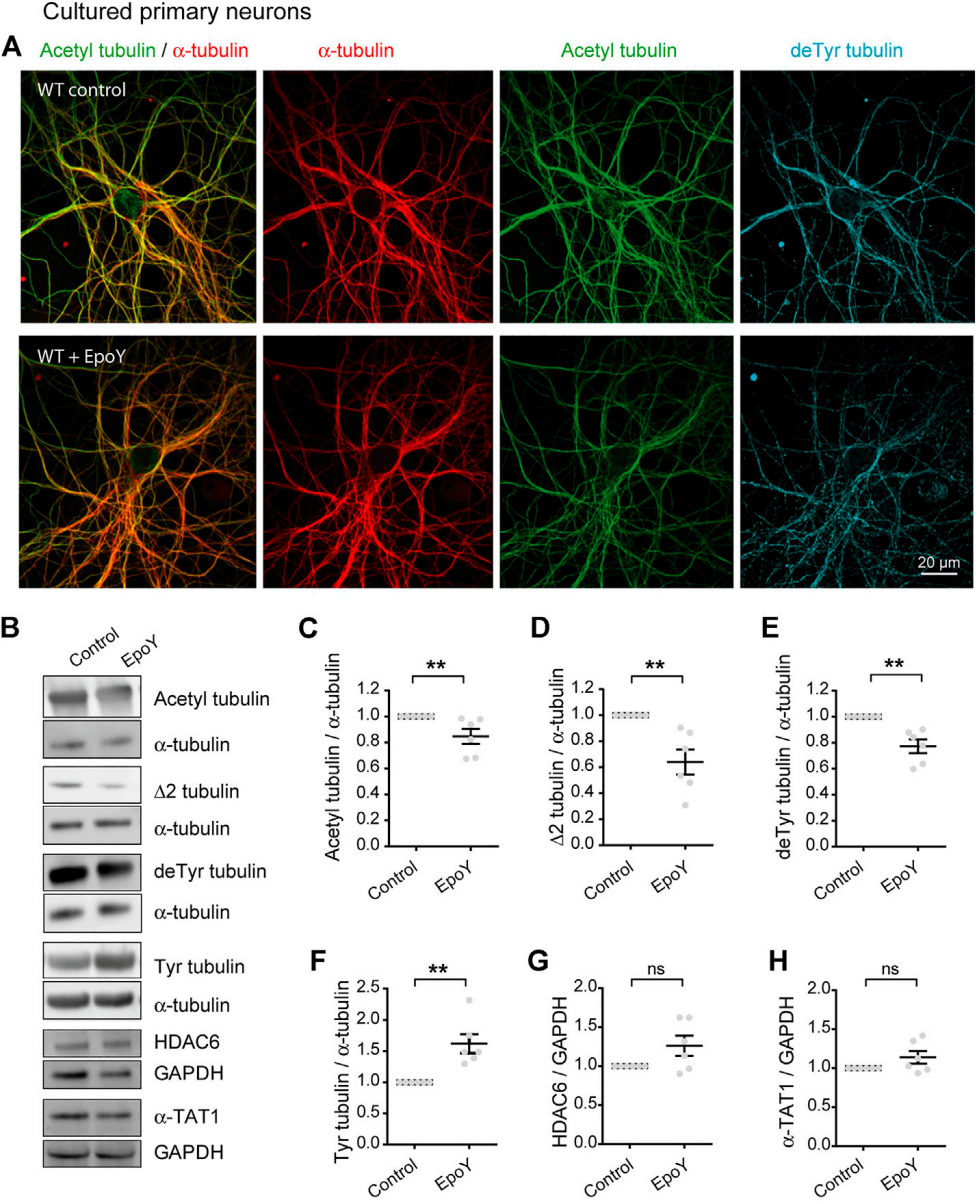
Decreased acetylated tubulin levels in cultured primary neurons with reduced detyrosination activity. **(A)** Confocal images showing representative examples of WT cultured hippocampal neurons at DIV17 treated or not with alkyne-epoY (VASH-SVBP inhibitor) at 5 µM over 12 h, stained for total α-tubulin (red), acetylated tubulin (acetyl, green), and detyrosinated tubulin (deTyr, blue). Scale bar: 20 µm.**(B)** Immunoblot analysis of acetylated tubulin, Δ2 tubulin, detyrosinated tubulin, tyrosinated tubulin, α tubulin, HDAC6, α-TAT1, and GAPDH from lysates of WT cultured cortical neurons at DIV15 treated or not with alkyne-epoY as in **(A)**. Immunoblot quantifications of the ratio of acetylated tubulin **(C)**, Δ2 tubulin **(D)**, detyrosinated tubulin **(E)** and tyrosinated tubulin **(F)** to total α-tubulin, and the ratio of HDAC6 **(G)** and α-TAT1 **(H)** to GAPDH. Data represent mean ± SEM. *n* = 6 independent neuronal differentiation experiments. Mann Whitney test, ns, not significant and **p < 0.01.

Collectively, these data indicate that the chronic absence of VASH-SVBP detyrosinating enzymes in SVBP KO neurons leads to a reduction in the proportion of acetylated MTs. To determine whether short-term inhibition of the VASH-SVBP enzyme likewise lowers the degree of tubulin acetylation, we treated wild-type neurons with the membrane-permeant protease inhibitor alkyne-epoY, which specifically inhibits the tubulin carboxypeptidase activity of VASH-SVBP (Aillaud et al., 2017). Immunofluorescence microscopy showed that a 12 h incubation with the inhibitor caused a noticeable weakening of both acetylated and detyrosinated MT fluorescence (Figure 4A). We then performed quantitative immunoblotting analysis of alkyne-epoY-treated cortical cultured neurons and found that VASH-SVBP inhibitor induced 15.27% + 5.82% decrease in the acetylated vs. total tubulin ratio (Figures 4B,C). This reduction accompanied a 22.72% + 5.23% drop in the proportion of detyrosinated and a 35.99% + 9.62% drop in Δ2 tubulin relative to total tubulin, and a 61.90% + 15.27% rise in that of tyrosinated tubulin (Figures 4B,D–F). Those changes were not correlated with variations in the amount of HDAC6 and α-TAT1 (Figures 4B,G,H, 126.10 + 12.86 and 114.00 + 8.15 for HDAC6 and α-TAT1, respectively).

Thus, the drop in acetylation can follow an acute reduction in detyrosination, consistent with the notion that the efficacy of MT acetylation can be decreased by loss of longer lived MTs.

## Discussion

Using a series of well-staged AD brains and age-matched controls, we recently showed that AD involves an increase in detyrosinated and Δ2 tubulins, two tubulin post-translational modifications associated with long-lived MTs (Peris et al., 2022). Herein we sought to determine whether AD affects MT acetylation, a post-translational modification that is also a marker of MT stability. Previous studies of MT acetylation in AD have yielded somewhat conflicting results (discussed further below). To address this issue, we first took advantage of a comparatively large set of AD brains and age-matched controls to assess anew the changes in acetylated tubulin that may occur during AD. The brains had been collected with a *post mortem* delay systematically shorter than 5 h, and well-defined areas were dissected for biochemical analysis, thereby minimizing the non-specific variability of human brain samples. With this number of subjects for each stage and of age-matched controls and repeating the analysis in four different brain regions for each subject, we could detect an early, persistent elevation of acetylated tubulin in diseased brains, and confirmed the significance of this observation in a linear mixed model. These biochemical results were validated by immunofluorescence on hippocampal sections from independently obtained control and AD individuals, showing that the average acetylated tubulin content was higher in AD neurons *in situ* than in controls, even in the neuron subgroup that had a relatively low content of hyper-phosphorylated Tau (i.e., presumably at an early stage of degeneration). Consistent with observations in AD subjects, hiPSC neurons bearing an APP London mutation sufficient to cause familial AD, which results in higher oAβ level and hyperphosphorylated Tau compared to isogenic control neurons, also produced more acetylated tubulin than isogenic WT controls.

Possible non-mutually exclusive explanations for the increase in acetylated tubulin include: 1) a simple increase in total tubulin; 2) a decrease in tubulin deacetylase levels or activity, or an augmentation of tubulin acetyl transferase levels or activity; 3) an increase in MT stability. In our biochemical analyses of the same samples, the relative amounts of total tubulin did not significantly differ between control and AD patients (Peris et al., 2022). However, while cytoplasmic deacetylase HDAC6 in AD brains did not change, in human iPSC APP-London neurons HDAC6 levels did increase without any change in α-TAT1 levels. This is not surprising since HDAC6 has long been known to be involved in cell stress responses and has a variety of targets besides tubulin (Kwon et al., 2007; LoPresti, 2020). Nevertheless, the increased amount of the deacetylase was not associated with reduced acetylated tubulin levels. Interestingly, raising the levels of detyrosinated MTs by depleting the re-tyrosination enzyme TTL, was sufficient to up-regulate acetylated tubulin in primary cultures of hippocampal and cortical neurons. In addition, as detyrosinated MTs are not subject to the depolymerizing activity of kinesin 13-type motors present in neurons (Peris et al., 2009), the enhanced detyrosination and loss of MT dynamicity observed in AD neurons (Peris et al., 2022) or primary neurons exposed to oligomeric Aβ (Qu et al., 2017) may have allowed the α-TAT1 more time to acetylate MTs despite its inherently slow catalytic rate (Szyk et al., 2014).

The present results stand in apparent contrast with some of the previous work. Immuno-histochemical observations by Hempen and Brion (1996) first indicated that neurons residing in the hippocampus of AD patients had low to undetectable amounts of acetylated tubulin, the level of which was inversely related to that of Tau immunoreactivity (Hempen and Brion, 1996). Since that pioneering study did not include control (non-demented) brain samples, and since the immunostaining images were not supported by biochemical analysis, it is hard to directly compare these data with ours. The discrepancy may arise from these authors using different immunodetection, microscopy, and quantitation methods, on a smaller series of subjects. We also note that all the subjects had reached an advanced disease stage (Braak V-VI) in which cellular reactions that are observable at earlier stages may become obscured by neurodegeneration. Post mortem intervals were also different from our present study, with known intervals ranging from 5.5 h in our study, to 22 h in theirs. Conversely, a more recent study reported that AD patients showed decreased tubulin levels along with increased acetylation in neurons containing neurofibrillary tau pathology (Perez et al., 2009). Similarly, Zhang et al. (2015) described that while the amount of both total tubulin and acetylated tubulin was reduced in AD brains, the proportion of acetylated tubulin was actually higher (Zhang et al., 2015). Even though their overall conclusion agrees with the present work, in our series of human samples we did not find any significant change in total tubulin content related to disease-progression (Peris et al., 2022). As the work of Perez et al. (2009) and Zhang et al. (2015) included only a fraction of AD subjects relative to our study and did not use Braak staging, their data cannot easily be compared to the present results.

Silva et al. (2017) reported that mitochondrial perturbations linked to AD caused hyper-activation of the protein deacetylase SIRT2, resulting in diminished levels of acetylated tubulin. While these findings were obtained by using a cellular system distinct from neurons (differentiated SHSY5Y neuroblastoma cells fused to platelets from AD patients), this study also reported a drop in acetylated tubulin in human AD brain samples and in cultured mouse neurons treated with synthetic Aβ for 24 h. However, the sample series used by Silva et al. (2017) was limited to five individuals in the control or AD conditions, all at Braak stages III-IV. Another *in vitro* study reported that tubulin acetylation went up after shorter Aβ exposure (Qu et al., 2017). The differences in Aβ effects on tubulin acetylation in cultured neurons may well be due to the use of vastly different Aβ concentrations (5 vs. 0.25 μM), its level of oligomerization, and the timing of exposure.

Consistent with our model and Qu et al. (2017) findings, Tsushima et al. (2015) reported that Aβ treatment increased tubulin acetylation also in cultured developing mouse neurons through a direct inhibitory effect of Aβ on HDAC6 activity (Tsushima et al., 2015). Besides Aβ, another inhibitor of HDAC6 activity is tau (Perez et al., 2009), providing another player in AD that limits HDAC6 activity. In the present work, we did not directly analyze HDAC6 nor ATAT1 activity, and so we cannot discard the possibility that less active HDAC6 or more active ATAT1 may contribute to tubulin hyperacetylation or allow higher HDAC6 levels to be seen concurrently with increased acetylated tubulin as in hiPSC APP London neurons. Indeed, tubulin acetylation due to an abundance of detyrosinated MTs could further accumulate when combined with a hyperactive ATAT1 or inefficient tubulin deacetylation caused by HDAC6 inhibition. These models are not mutually exclusive and further work is necessary to detail the mechanisms underlying these possibilities.

The notion that AD neurons contain an elevated proportion of stable MTs speaks to core pathological mechanisms and possible therapeutic options. A long-prevailing view has held that AD involved a deficit in neuronal MT stabilization, due to the hyper-phosphorylation and aggregation of Tau, which disrupts its ability to bind and bundle MTs (Weingarten et al., 1975; Kanai et al., 1992). Hence, it has been proposed that MT-stabilizing anti-cancer drugs, such as paclitaxel and Epothilone D, could be re-positioned as a compensatory treatment for AD; but so far clinical trials have been unsuccessful (Brunden et al., 2012; Varidaki et al., 2018; Hoskin et al., 2019; Tsai et al., 2020). However, current research unexpectedly revealed that Tau preferentially binds to dynamic MTs, allowing them to sustain longer labile regions in the axon (Qiang et al., 2018). Furthermore, various Tau isoforms and phosphorylation states differ in their effects on MT networks (Prezel et al., 2018). Thus, early cellular AD pathology may lead to an excess rather than a loss of stable neuronal MTs, and restoration of a dynamic MT pool, bringing, e.g., resilience to dendritic spines (Peris et al., 2022) may provide a better avenue for possible MT-based AD therapies.

The consequences of accumulating acetylated tubulin in AD may be multifold: 1) an increase in acetylated MTs may affect the binding of kinesin and dynein to MTs, leading to remapping of MT-dependent transport and organelle distribution (Reed et al., 2006; Alper et al., 2014; Zhang J. et al., 2014; Balabanian et al., 2017; Farias et al., 2017); 2) hyperacetylated MTs may drive mitochondrial fission by promoting Drp1 activation and mitochondrial translocation under stress conditions (Perdiz et al., 2017); 3) tubulin hyperacetylation may on its own affect the turnover of MT polymer and further induce abnormal MT longevity (Portran et al., 2017; Xu et al., 2017; Eshun-Wilson et al., 2019). Regardless of the dominant pathophysiological mechanism, this study confirms that loss of MT dynamicity and accumulation of tubulin PTMs associated with an increase in MT stability underlies both sporadic and familial AD, indicating that it has become necessary to consider also targeting these changes to prevent or restore cognitive decline in AD.

### Beyond Alzheimer’s disease: A novel crosstalk between acetylation and detyrosination of tubulin

Our results show that MT acetylation can be affected by the tubulin tyrosination/detyrosination cycle. Whether this occurs because stable detyrosinated MTs are an easier target for α-TAT1 but a worse substrate for HDAC6 remains to be determined. Nevertheless, our study provides groundbreaking evidence that an increase in detyrosinated and/or Δ2 tubulins is able to influence a different kind of tubulin modification, lys40 acetylation, driving a positive feedback cycle in the modification of the MT. Interestingly, this relationship seems to be reciprocal, as observed by the decreased tubulin detyrosination in α-TAT1 depleted cells (Xu et al., 2017), suggesting that a functional interplay between these two tubulin PTMs may have a critical role in the regulation of MT function. Indeed, while this crosstalk may play a physiological role that has yet to be elucidated, in pathological conditions such as AD, it may lead to undesirable cellular consequences caused by an overly modified and less dynamic MT cytoskeleton.

## Materials and methods

### Animals

All experiments involving mice were conducted in accordance with the policy of the Institut des Neurosciences de Grenoble (GIN) and in compliance with the French legislation and European Union Directive of 22 September 2010 (2010/63/UE). Mice homozygous for an inactivated tubulin tyrosine ligase allele (TTL KO) were obtained as previously described (Erck et al., 2005). Mice homozygous for an inactivated Small Vasohibin Binding Protein allele (SVBP KO) were obtained as previously described (Pagnamenta et al., 2019). All experiments involving rats were approved by the Committee on the Ethics of Animal Experiments of Columbia University and performed according to Guide for the Care and Use of Laboratory Animals distributed by the National Institutes of Health. E18 pregnant Sprague Dawley rats were purchased from Charles River Laboratories.

### Biochemical analysis of post-mortem human brain tissues

Human brains were provided by the Human Brain Tissue Bank, Semmelweis University, Budapest, Hungary. Tissue samples consist of four regions of the brain (entorhinal cortex, hippocampus, temporal and lateral prefrontal cortex) coming from a panel of 29 male and female patients aged from 52 to 93 years: 11 controls, 5, 6, and 7 from each group corresponding to Braak stadium I-II, III-IV, and IV-V (Supplementary Table S1).

#### Extraction

Brain samples were homogenized 2 × 30 s at room temperature in (10% w/vol) 10 mM Tris, 0.32 M sucrose, pH 7.4 containing complete inhibitors cocktail (Roche) using ready to use Precellys Lysing Kit (Bertin Technologies) in a Minilys apparatus. After lysis, the homogenates were collected, frozen in liquid nitrogen and then stored at −80°C until use. When needed, frozen aliquots were diluted v/v with RIPA buffer (50 mM Tris, 150 mM NaCl, 1% NP40, 0.5% deoxycholate, 0.1% SDS, pH = 8) stirred 30 min at 4°C and then centrifuged 10 min at 14,000 g at 4°C. Supernatants were frozen in liquid nitrogen and then stored at −80°C until use.

#### Antibodies

Mouse monoclonal anti acetylated tubulin antibody (6-11B-1, T7451) was from SIGMA, mouse monoclonal anti HDAC6 (D-11, sc-28386) was from Santa Cruz, mouse monoclonal anti α-tubulin antibody (α3A1) was described in Paturle-Lafanechere et al. (1994).

#### Western blot analysis and quantification

RIPA supernatants (10 µl) were subjected to electrophoresis on stain free 4%–15% gels (Bio Rad) and then quickly transferred to Nitrocellulose using Trans-Blot Turbo Transfer System (Bio Rad). Proteins on the membrane were revealed using specific antibodies against acetylated and α tubulin. Anti Acetylated-Tub (1/10,000) and anti α-tubulin (1/10,000) antibodies were used with the appropriate peroxidase-/labeled secondary antibodies. Secondary antibody signal was revealed using Pierce ECL Western blotting substrate (Thermo scientific) and analyzed with ChemiDoc™MP Imaging System (Bio Rad) using Image Lab software (stain free gel and chemoluminescence protocol) for quantification. For each lane of the blot, the software measures the integrated volume of the band corresponding to the antigen of interest. The signal is then normalized according to the total protein measured in the same lane. For every blot, one lane is dedicated to an internal standard corresponding to a WT sample (used for the entire study) and the protein-normalized signal of this standard is considered as 100%, therefore each unknown sample is calculated as a % of this standard. For each brain sample, three independent blots were performed and the mean intensity was calculated.

### Immunohistochemical analysis of *post-mortem* brain tissues

De-identified human autopsy brain tissue was obtained from the New York Brain Bank at Columbia University (New York, NY, United States). Neuropathologically-confirmed Alzheimer’s disease cases and controls were processed following published protocols (Vonsattel et al., 2008).

#### Immunolabelling

Brain paraffin blocks were cut into 5 μm sections and deparaffinized in xylene (7 min twice) followed by 95% ethanol, 90% ethanol, 80% ethanol, and 70% ethanol; 5 min each). After washing the slices in distilled H_2_O three times, citric acid was used to retrieve antigen by boiling samples for 15 min. Sections were cooled for 15 min, washed three times with PBS and blocked with serum for 1 h at room temperature prior to staining with primary antibodies (anti-acetylated tubulin, 1/100 and T212 anti Tau, 1/500) at 4°C overnight. The next morning sections were washed three times with PBS and stained with appropriate secondary antibodies (Cy3 donkey anti mouse, 1/200; Alexa 488 donkey anti rabbit, 1/200; DAPI, 1/1,000) for 1 h at room temperature. Stained samples were washed three times with PBS and incubated in 0.1% black Sudan in 70% ethanol for 5 min to reduce auto-fluorescence of lipofuscin, rinsed with 70% ethanol until black was gone and rehydrated in distilled H_2_O.

#### Image acquisition and analyses

Coverslips were mounted with Fluoromount prior to imaging using an Oympus VS-ASW FL 2.7 (Build 11032) slide scanner and Olympus soft imaging solutions camera XM10. Images were taken using a ×10 objective and same exposure time was used for the same primary antibody (acetylated tubulin: 100 ms; T212 tau: 10 ms; 4′,6-diamidino-2-phenylindole: 10 ms). The images were converted into Tiff files for analysis using MetaMorph software. Pyramidal neuron cell bodies and proximal dendrites were randomly selected in the anterior hippocampal formation and average fluorescence intensity was measured for acetylated tubulin, as well as for T212. An average of 150 neurons were selected for each case. Pyramidal neurons were arbitrarily classified into low T212 (1–600 A.U.), intermediate T212 (600.01–1,400 A.U.) and high T212 (1,400.01–3,400 A.U.) based on T212 staining intensity in the cell body.

### Mutant APP and isogenic control human induced pluripotent stem cell maintenance and differentiation

Human induced pluripotent stem cells (hiPSCs) in which the APPV717I (London) mutation was knocked into one allele of the control IMR90 cl.4 iPSC line (WiCell; (Yu et al., 2007; Yu et al., 2009; Hu et al., 2011); using CRISPR/Cas9 was generated by Dr. Andrew Sproul’s lab, as has been described previously (Sun et al., 2019).

#### Maintenance

APPLondon knock-in (cl. 88) and the isogenic parent line were maintained feeder-free in StemFlex media (Life) and Cultrex substrate (Biotechne).

#### Neuronal differentiation

Bankable neural progenitors were first generated using manual rosette selection and maintained on Matrigel (Corning) as has been described previously (Topol et al., 2015; Sun et al., 2019). Terminal differentiations were carried out by plating 165,000–185,000 NPCs per 12 well plate in N2/B27 media (DMEM/F12 base) supplemented with brain-derived neurotrophic factor (20 ng/ml; Biotechne) and laminin (1 µg/ml; Biotechne) on PEI (0.1%; Sigma)/laminin (20 µg/ml)-coated plates. After 1 week of differentiation, 100 nM Cytosine β-D-arabinofuranoside hydrochloride (Sigma) was added to reduce proliferation of remaining neural progenitors. A similar strategy was used for imaging plates (MaTek Lifesciences). Differentiations were analyzed 30–40 days post plating. For later passage of neural progenitors, we employed a CD271-/CD133+/CD184+ (Biolegend) flow-cytometry purification strategy to remove minority neural crest contaminants (CD271+) that can expand over time, as previously done. Western blot analyses of reprogrammed cortical neurons: Cell lysates from WT and APP mutant human cortical neurons at 30–40 days of differentiation were lysed in Laemmli sample buffer and boiled at 96°C for 5 min. Cell lysates were sonicated by probe sonication to shear cellular debris and genomic DNA. Proteins were separated by 4%–12% Bis-Tris gel (Invitrogen) and transferred to nitrocellulose membrane. After blocking in 5% BSA/TBS, membranes were incubated with primary antibodies (anti total tau (tau 46) (sc-32274) from Santa Cruz; anti tau AT8 (MN1020 and 30505) from Invitrogen and Cell Signaling Technology, and anti-GAPDH (MA5-15738 and PA5-85074) from Invitrogen; anti acetylated tubulin antibody (T7451) from Sigma; anti α-tubulin antibody (11224-1-AP) from Proteintech; HDAC6 (12834-1-AP) from Proteintech; anti α-TAT1 (BS-9535R) from Bioss) at 4°C overnight or 2 h at room temperature. Membranes were subsequently washed three times for 10 min in TBST, incubated for 1 h with appropriate secondary antibodies (LI-COR Biosciences), and then washed again. When needed, membranes were stripped with Restore™ Western Blot Stripping Buffer (ThermoFisher). Image acquisition was performed with an Odyssey infrared imaging system (LI-COR Biosciences) and analyzed with ImageJ.

### Primary neuronal cultures

#### Rat hippocampal neurons

Rat hippocampi were dissected from E18 embryos, and neurons were plated on 100 µg/ml poly-D-lysine–coated 12-well plates at the density of 3 × 10^5^ cells/well for biochemistry assays. Primary neurons were maintained in Neurobasal medium (Invitrogen) with the supplement of 2% B-27 (Invitrogen) and 0.5 mM glutamine (Invitrogen), and one third of medium was changed every 3–4 days up to 4 weeks in culture.

##### Lentivirus infection

To induce acute tubulin tyrosine ligase reduction, hippocampal neurons from WT rat embryos were infected at 17 days *in vitro* (DIV) with lentiviral vectors containing either control or tubulin tyrosine ligase-targeting shRNA, and incubated until DIV 21.

##### Western blot analyses of primary hippocampal neurons

21 DIV rat hippocampal neurons were lysed in Laemmli sample buffer and boiled at 96°C for 5 min. The procedure continued as described above for the reprogrammed cortical neurons, plus using the primary antibody anti tubulin tyrosine ligase (13618-1-AP) from Proteintech.

#### Mouse hippocampal/cortical neurons

Mouse hippocampi/cortices were dissected from embryos (E18.5) and digested in 0.25% trypsin in Hanks’ balanced salt solution (HBSS, Invitrogen, France) at 37°C for 15 min. After manual dissociation, hippocampal neurons used for immunostaining were plated at a concentration of 5,000–15,000 cells/cm^2^ on 1 mg/ml poly-L-lysine-coated coverslips. Cortical neurons used for immunoblot analysis were plated at a concentration of 100,000 cells/cm^2^ on 1 mg/ml poly-L-lysine-coated dishes. Neurons were incubated 2 h in DMEM-10% horse serum and then changed to MACS neuro medium (MiltenylBiotec) with B27 supplement (Invitrogen, France).

##### Antibodies

In addition to the ones used in the post-mortem brain analysis, rabbit polyclonal anti α-TAT1 (clinisciences, bs-9535R), and rabbit polyclonal anti GAPDH (sigma Aldrich, rG9545-200µl). Δ2 tubulin, tyrosinated, detyrosinated and total α-tubulin were described in Aillaud et al. (2017).

##### For immunostaining

Hippocampal neurons (15 DIV) were fixed with 4% paraformaldehyde and 4% sucrose in PBS for 20 min and permeabilized with 0.25% Triton X-100/PBS for 5 min. Fixed cells were then incubated with primary antibodies α-tubulin (1/1,000), acetylated α-tubulin (1/2,000) and detyrosinated α-tubulin (1/1,000) for 3 h in 0.1% PBS/Tween and then with appropriate fluorophore-conjugated secondary antibodies for 1 h at room temperature.

##### Confocal imaging

Fluorescent images were acquired with a ×63 oil immersion objective (1.4 NA) using a spinning disk ZEISS Axio Observer Z1/ROPER confocal microscope coupled to a LiveSR module to improve the resolution.

##### Western blot analyses of primary cortical neurons

Cortical neurons (15 DIV) lysed in Laemmli buffer and boiled at 96°C for 5 min. The protein contents were revealed using specific antibodies against acetylated α-tubulin (1/10,000), Δ2 tubulin (1/10,000), detyrosinated α-tubulin (1/8,000), tyrosinated α-tubulin (1/5,000), total α-tubulin (1/8,000), HDAC6 (1/500), α-TAT1 (1/1,000), and GAPDH (1/10,000). And analyzed by quantitative western blot with the protocol used for human brain samples as described above, but using the appropriate fluorescent, either A488 or Cy5-labeled, secondary antibodies. Several neuronal cultures were used as indicated in figure legends and for each sample, three independent blots were performed.

### Plasmids

For tubulin tyrosine ligase expression, a lentiviral vector (Addgene #12255, kind gift from D. Trono) was used to express cDNA encoding human tubulin tyrosine ligase (NP_714923, Origene #RC207805L2) as described (Peris et al., 2022). For lentiviral shRNA expression, a tubulin tyrosine ligase shRNA sequence, cloned in pLKO.1 vector, was purchased from Sigma-Aldrich: shTTL (TRCN0000191515, sequence: 5′—CCG GCA TTC AGA AA GAG TAC TCA ACT CGA GTT GAC TAC TCT TTC TGA ATG CTT TTT TG′—3′). The SHC001 pLKO.1-puro Empty Vector (Sigma) was used as control (shControl).

### Lentivirus production

Lentiviral particles were produced using the second-generation packaging system as previously described (Peris et al., 2022). Lentivirus encoding tubulin tyrosine ligase cDNA (packaging vectors, pWPT-based vector, Addgene, Cambridge, MA) and shTTL and control shRNA (packaging vectors pLP1, pLP2, and pLP-VSV-G, Thermofisher) were produced by co-transfection with the psPAX2 and pCMV-VSV-G helper plasmids, into HEK293T cells obtained from ATCC (ATCC-CRL-3216) using the calcium phosphate transfection method. Viral particles were collected 48 h after transfection by ultra-speed centrifugation, prior to aliquoting and storage at −80°C.

### Statistical analysis

Data analyses, statistical comparisons, and graphs were generated using GraphPad prism or the R programming language. Statistical analysis of differences between two groups was performed using Student’s *t* tests for populations with Gaussian distribution or else with Mann Whitney’s test. For Figure 1B and Supplementary Figure S1B, as regular two-way ANOVA was not suitable we used a linear mixed model and calculation of model coefficients by restricted maximum likelihood estimation (using the R lmer package). The significance of fixed effects (Braak stage and brain region) was then evaluated by Wald type II F tests (with Kenward-Roger correction) of the null hypothesis for each of the model coefficients. Post hoc comparisons were performed between non-weighted marginal means, using Sidak tests. The calculations were performed with the R car and emmeans packages. For Figure 1E, the null hypothesis of no difference between the three groups was tested with the Kruskal-Wallis test. Mean differences were considered significant at *p* < 0.05 (*p < 0.05; **p < 0.01; ***p < 0.001 and ****p < 0.0001).

## Data Availability

The raw data supporting the conclusions of this article will be made available by the authors, without undue reservation.

## Abbreviations

MT, microtubule; AD, Alzheimer’s disease; TTL, tubulin tyrosine ligase; VASH1, vasohibin 1; VASH2, vasohibin 2; SVBP, small vasohibin binding protein; SVBP KO, small vasohibin binding protein knock-out; TTL KO, tubulin tyrosine ligase knock-out; WT, Wild-Type; iPSCs, induced pluripotent stem cells; HDAC6, histone deacetylase 6; α-TAT1, α-tubulin acetyltransferase 1.
