# Supplementary material for: Crosstalk between acetylation and the tyrosination/detyrosination cycle of α-tubulin in Alzheimer’s disease
Source: Front Cell Dev Biol. 2022 Aug 26;10:926914. doi: 10.3389/fcell.2022.926914 (PMC9459041; doi:10.3389/fcell.2022.926914)
Supplement: Supplementary file 1 [file Table1.DOCX]

**Supplementary Information**

| **Control patients** | | | | |
| --- | --- | --- | --- | --- |
| **Patient Number** | **Neuropathological disorder** | **Gender** | **Age** | **Cause of death** |
| **#61** | Control | male | 65 | pulmonary embolism, arterosclerosis, heart failure |
| **#199** | Control | female | 76 | heart failure |
| **#223** | Control | male | 61 | heart failure |
| **#220** | Control | male | 63 | pulmonary embolism |
| **#164** | Control | male | 85 | acute cardiorespiratoric insufficiency |
| **#198** | Control | female | 68 | parieto-occipital and frontal stroke, pneumonia, respiratory insufficiency |
| **#205** | Control | male | 66 | cardiovascular-pulmonary insufficiency, acute lymphoid leukemia |
| **#217** | Control | male | 60 | acute myocardial infarction |
| **#266** | Control | male | 61 | vertebrobasilar stroke (right side) |
| **#269** | Control | male | 79 | stroke in the left cortical hemisphere, herniatio |
| **#276** | Control | female | 79 | stroke, pneumonia |
| **Alzheimer's disease patients** | | | | |
| **#184** | Braak stadium I - II | male | 83 | emolitio cerebri |
| **#187** | Braak stadium I - II | male | 62 | respiratory and cardiac insufficiency |
| **#191** | Braak stadium I - II | female | 93 | Alzheimer’s disease, cardiovascular-respiratory insufficiency |
| **#218** | Braak stadium I - II | female | 72 | acute cardiac insufficiency |
| **#219** | Braak stadium I - II | male | 67 | pulmonary embolism |
| **#185** | Braak stadium III - IV | male | 80 | stroke (right side), herniatio |
| **#196** | Braak stadium III - IV | female | 78 | stroke, arteria cerebri media, brain hemorrhage |
| **#230** | Braak stadium III - IV | female | 79 | pulmonary embolism |
| **#197** | Braak stadium III - IV | male | 64 | myocardial infarction |
| **#267** | Braak stadium III - IV | female | 91 | stroke, arteria cerebri media (left side) |
| **#279** | Braak stadium III - IV | male | 79 | stroke (right side) |
| **#154** | Braak stadium V-VI | female | 72 | acute myocardial infarction, earlier heart failure, arterosclerosis |
| **#167** | Braak stadium V-VI | female | 65 | suicide (hanging - asphyxia) |
| **#195** | Braak stadium V-VI | male | 83 | respiratory and cardiac insufficiency |
| **#202** | Braak stadium V-VI | male | 84 | cardiac and respiratory insufficiency |
| **#212** | Braak stadium V-VI | female | 87 | dementia, myocardial insufficiency |
| **#229** | Braak stadium V-VI | female | 78 | Alzheimer’s disease |
| **#232** | Braak stadium V-VI | female | 77 | cardiorespiratory insufficiency |

**Table S1. Details of control and Alzheimer’s disease patients classified into Braak stages as shown in Figure 1 A-B and S1 A-B.** The age, gender and cause of death are listed for each patient. Post-mortem interval (PMI) between the time of death and the collection of tissues is inferior of 300 minutes.
