# Supplementary figures and images for: Crosstalk between acetylation and the tyrosination/detyrosination cycle of α-tubulin in Alzheimer’s disease
Source: Front Cell Dev Biol. 2022 Aug 26;10:926914. doi: 10.3389/fcell.2022.926914 (PMC9459041; doi:10.3389/fcell.2022.926914)

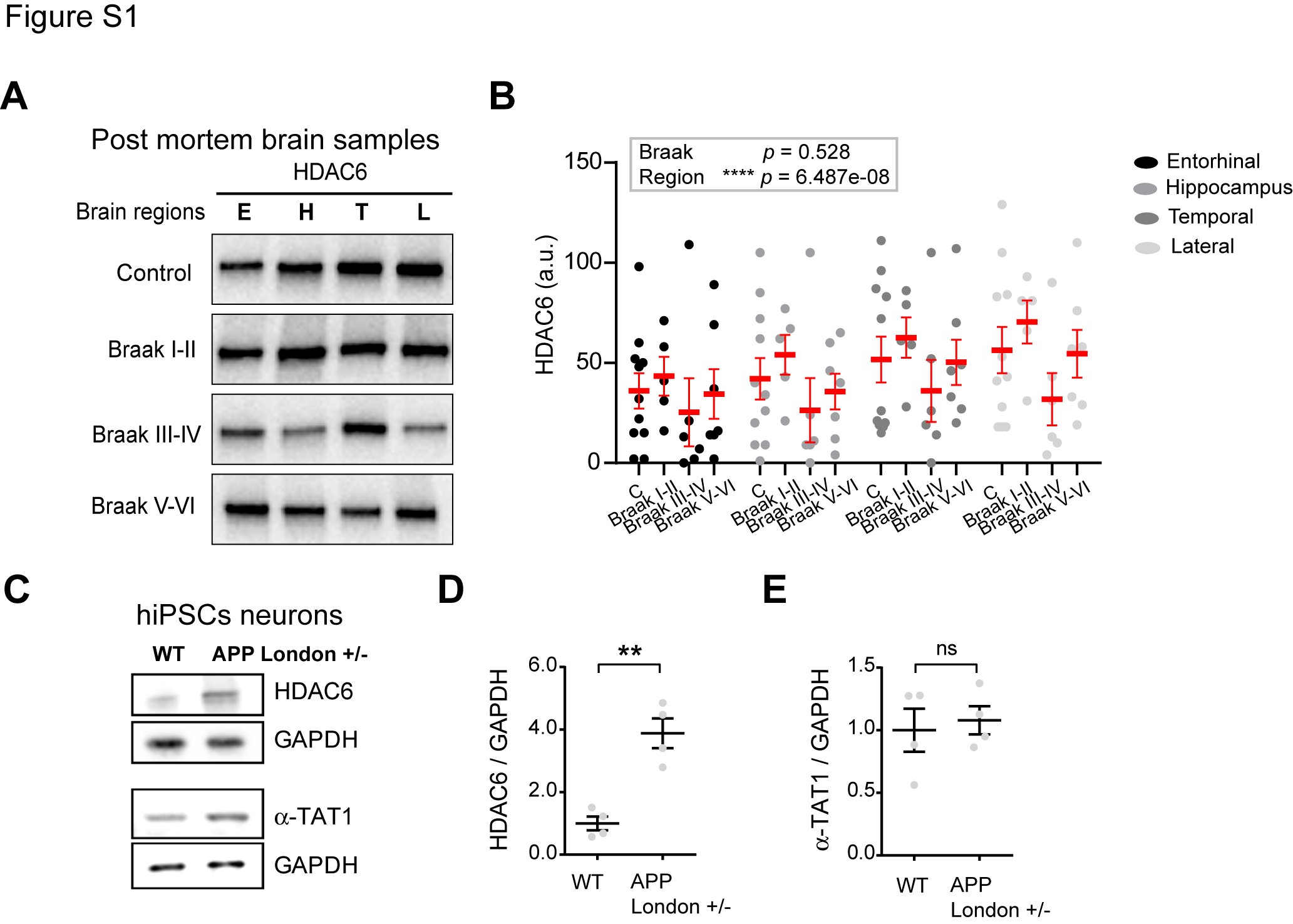

Supplement: Supplementary file 2 [file Image1.JPEG]
