# Supplementary material for: Crosstalk between acetylation and the tyrosination/detyrosination cycle of α-tubulin in Alzheimer’s disease
Source: Front Cell Dev Biol. 2022 Aug 26;10:926914. doi: 10.3389/fcell.2022.926914 (PMC9459041; doi:10.3389/fcell.2022.926914)
Supplement: Supplementary file 3 [file Table2.DOCX]

**Supplementary Information**

| Area | Control (95% C.I.) | Braak V-VI (95% C.I.) | p value (Dunnett) |
| --- | --- | --- | --- |
| Entorhinal cortex | 0.514 0.915 | 1.144 1.647 | 0.0003 |
| Hippocampus | 0.530 0.931 | 1.063 1.566 | 0.0018 |
| Lateral prefrontal cortex | 0.485 0.886 | 0.928 1.431 | 0.00094 |
| Temporal cortex | 0.448 0.849 | 0.973 1.476 | 0.0021 |

**Table S2**. 95% confidence intervals (C.I.) for acetylated to total tubulin ratios in four brain areas from control and advanced (Braak V-VI) AD subjects. P-values for AD vs. control comparisons are also indicated (post hoc Dunnett test with Kenward - Roger correction).
