# Supplementary material for: Crosstalk between acetylation and the tyrosination/detyrosination cycle of α-tubulin in Alzheimer’s disease
Source: Front Cell Dev Biol. 2022 Aug 26;10:926914. doi: 10.3389/fcell.2022.926914 (PMC9459041; doi:10.3389/fcell.2022.926914)
Supplement: Supplementary file 4 [file Table3.DOCX]

**Supplementary Information**

| **Post #** | MP N08-17 | MP N03-166 | MP N09-270 | MP N07-47 | OC04-18 | MP N03-161 |
| --- | --- | --- | --- | --- | --- | --- |
| **classification** | Control | Control | Control | AD | AD | AD |
| **Age** | 74 | 87 | 90 | 75 | 82 | 86 |
| **Sex** | M | M | M | M | M | M |
| **Braak NFT stage** | 3 | 3 | 3 | 6 | 6 | 6 |
| **CERAD plaque score** | none | sparse | sparse | frequent | frequent | frequent |
| **Amyloid angiopathy** | 0 | 0 | 0 | + | + | + |
| **NIAR** | 0 | Low | Low | High | High | High |
| **PMI (min)** | 93 | 310 | 270 | 295 | 127 | 260 |

**Table S3. Table of case descriptions of postmortem human Alzheimer’s disease and control brains shown in Figure 1 C-E.** The age and gender are listed for each patient. The Braak NFT stage, CERAD plaque score and amyloid angiopathy are listed to demonstrate the pathological hallmarks of Alzheimer Disease present in each brain sample. The NIA-Reagan score (NIAR) is a post-mortem diagnosis score of the likelihood of having Alzheimer’s disease, which considers the Braak stage and CERAD score. Post-mortem interval (PMI) between the time of death and the collection of tissues is inferior of 300 minutes.
