# Supplementary material for: Crosstalk between acetylation and the tyrosination/detyrosination cycle of α-tubulin in Alzheimer’s disease
Source: Front Cell Dev Biol. 2022 Aug 26;10:926914. doi: 10.3389/fcell.2022.926914 (PMC9459041; doi:10.3389/fcell.2022.926914)
Supplement: Supplementary file 5 [file DataSheet1.DOCX]

**Supplementary Information**

**Figure S1:** HDAC6 and α-TAT1 levels in sporadic and familial form of AD (**A**) Representative immunoblots of HDAC6 levels in brain homogenates from entorhinal cortex (E), hippocampus (H), temporal (T) and lateral prefrontal cortex (L) from control, early AD (Braak I-II), middle AD (Braak III-IV) and late AD (Braak V-VI) patients. (**B**) Quantification of HDAC6 in each brain region from control and Alzheimer’s disease patients. An internal standard corresponding to a WT sample was used for normalization and considered as 100% and the values for each unknown sample was calculated as a % of this standard (see material and methods). Each dot in the graph represents the mean of a triplicate analysis of one sample. The mean ± S.E.M. of these dot values is shown for each factor combination. The dependence of protein levels on, respectively, clinical stage and brain area was quantitated in each case using a linear mixed model, with Braak stage and brain region as fixed effect factors. Boxed p values measure the overall significance of these factors (type II Wald F test of model coefficients). In each brain area, post-hoc testing of variations due to individual Braak stages was performed by Dunnett’s test of differences with control showing no significant difference. n=11, 5, 6 and 7 brain samples for control, Braak I-II, Braak III-IV and Braak V-VI respectively. (**C**) Immunoblot analysis of HDAC6, α-TAT1 and GAPDH from lysates of DIV 30 - 40 human cortical neurons, derived from WT and APP London mutation (V717I) knocked-in hiPSCs isogenic lines. GAPDH was used for normalization. Immunoblot quantifications of HDAC6 (**D**) and α-TAT1 (**E**). Graphs represent mean ± SEM. n = 4 independent neuronal differentiation experiments. Unpaired t-test, ns = not significant, ** p < 0.01.
